# RNF187 Facilitates Proliferation and Migration of Human Spermatogonial Stem Cells Through WDR77 Polyubiquitination

**DOI:** 10.1111/cpr.70042

**Published:** 2025-04-08

**Authors:** Haoyue Hu, Xiaoxue Xi, Bing Jiang, Kehan Wang, Tiantian Wu, Xia Chen, Yueshuai Guo, Tao Zhou, Xiaoyan Huang, Jun Yu, Tingting Gao, Yibo Wu, Bo Zheng

**Affiliations:** ^1^ Human Reproductive and Genetic Center Affiliated Hospital of Jiangnan University Wuxi China; ^2^ State Key Laboratory of Reproductive Medicine and Offspring Health, Center for Reproduction and Genetics, the Affiliated Suzhou Hospital of Nanjing Medical University, Suzhou Municipal Hospital, Gusu School Nanjing Medical University Suzhou China; ^3^ Center of Reproductive Medicine, Department of Obstetrics and Gynecology Affiliated Hospital of Nantong University Nantong China; ^4^ State Key Laboratory of Reproductive Medicine and Offspring Health, Department of Histology and Embryology, School of Basic Medical Sciences Nanjing Medical University Nanjing China; ^5^ Research Institute for Reproductive Medicine and Genetic Diseases Wuxi Maternity and Child Health Care Hospital Wuxi China; ^6^ Institute of Reproductive Medicine, Medical School of Nantong University Nantong University Nantong China; ^7^ Department of Reproductive Medicine Center, Changzhou Maternal and Child Health Care Hospital, Changzhou Medical Center Nanjing Medical University Changzhou China

**Keywords:** EGR1, RNF187, spermatogonial stem cells, ubiquitin, WDR77

## Abstract

The E3 ubiquitin ligase RNF187, also known as RING domain AP1 coactivator‐1, is a member of the RING finger family. RNF187 is indispensable for the proliferation and migration of GC‐1 cells derived from mouse spermatogonia and GC‐2 cells derived from spermatocytes. However, it remains unclear whether RNF187 plays a crucial role in the self‐renewal and migration of human spermatogonial stem cells (SSCs). In this study, we observed a positive correlation between RNF187 expression and the proliferation and migration of human SSCs. Through co‐immunoprecipitation and mass spectrometry analyses, we identified WD repeat‐containing protein 77 (WDR77) as an interacting partner of RNF187. Specifically, RNF187 recognises the K118 site of WDR77 through lysine 48‐linked polyubiquitination, subsequently mediating its degradation via the ubiquitin‐proteasome system (UPS). Further studies have revealed that decreased expression of WDR77 diminishes the symmetric dimethylation at H4R3 (H4R3me2s) catalysed by its interacting protein, the arginine methyltransferase PRMT5. This, in turn, relieves the transcriptional repression of early growth response protein 1 (EGR1), a positive regulator for human SSC maintenance. In conclusion, this study has unveiled a pivotal role for RNF187 in the proliferation and migration of human SSCs. This may provide a promising strategy for addressing non‐obstructive azoospermia (NOA) caused by SSC dysfunction.

## Graphical abstract

RNF187 mediates the polyubiquitination and subsequent degradation of WDR77, reduces H4R3me2s catalysed by the WDR77/PRMT5 complex, and derepresses the transcription of *EGR1*, thereby promoting the proliferation and migration of human SSCs.

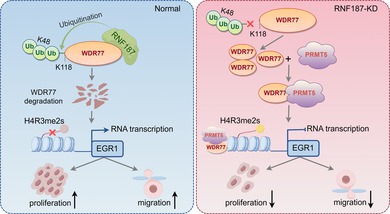

## 1 Introduction

Male factor infertility contributes to 50% of infertility cases globally, with the most severe form being non‐obstructive azoospermia (NOA), which affects approximately 10%–15% of infertile men [1]. Spermatogonial stem cells (SSCs) are the primary source of spermatogenesis, maintaining continuous sperm production in adulthood through self‐renewal and differentiation into sperm [2, 3, 4]. Previous studies using knockout mouse models have demonstrated that defects in SSCs self‐renewal are a significant contributor to the development of NOA [5, 6, 7]. However, due to interspecies differences and other confounding factors, the findings from mouse models have limited translational relevance to humans.

Ubiquitination is one of the most common post‐translational modifications and is widely involved in cellular biological processes [8, 9]. This modification is catalysed by a series of enzymatic reactions involving the ubiquitin‐activating enzyme (E1), ubiquitin‐conjugating enzyme (E2), and ubiquitin ligase (E3) [10, 11]. The ubiquitin‐proteasome system (UPS) plays a critical role in the precise degradation of misfolded or damaged proteins, thereby preventing the excessive accumulation of dysfunctional proteins that could impair cellular functions [12, 13]. Its dysfunction is associated with the development of a variety of diseases, particularly in the pathogenesis of NOA [14].

RING finger protein 187 (RNF187), also known as RING domain AP‐1 co‐activator‐1 (RACO‐1), is a RING domain‐containing ubiquitin E3 ligase [15]. RNF187 was initially identified as a substrate of the arginine methyltransferase PRMT1, which methylates two arginine residues on RNF187 [16]. This methylation induces a conformational change that stabilises RNF187 through the formation of K63‐linked ubiquitin chains, promoting its dimerization and interaction with c‐Jun, ultimately enhancing tumour cell proliferation [15]. Previous studies have shown that upregulation of RNF187 induces epithelial‐to‐mesenchymal transition (EMT) in hepatocellular carcinoma and non‐small‐cell lung cancer cells [17, 18, 19]. RNF187 is also associated with the progression of osteosarcoma and triple‐negative breast cancer [20, 21]. However, the role of RNF187 in human SSCs is still unclear.

WD repeat‐containing protein 77 (WDR77), a key member of the WD repeat protein family, plays a crucial role in biological processes such as cell cycle regulation, epigenetic modulation of gene expression, and DNA damage repair [22, 23]. WDR77 is essential during lung development, and it is reactivated during lung cancer progression, promoting the growth of tumour cells [24]. WDR77 markedly enhances the methyltransferase activity of PRMT5 by forming a complex with it, thereby jointly promoting the proliferation of squamous cell carcinoma via the ΔNp63α‐p21 signalling pathway [25]. In the pathogenesis of hepatitis B, the hepatitis B virus upregulates the E3 ubiquitin ligase DDB1, which promotes the degradation of WDR77. Consequently, the diminished activity of the PRMT5/WDR77 complex leads to increased transcription of covalently closed circular DNA (cccDNA), thereby facilitating the progression of hepatitis B [26]. Furthermore, a study indicates that the WDR77/PRMT5 complex plays a pivotal role in the development of the human fetal testis [27]. However, the specific functions and underlying molecular mechanisms of WDR77 within the male reproductive system, particularly in SSCs, remain largely elusive to date.

Understanding the role of RNF187 in human SSCs is critical due to fundamental interspecies differences that limit the translational relevance of mouse models. While mouse SSCs rely on POU5F1 for self‐renewal, this marker is absent in human SSCs, which instead utilise UTF1 and FGFR3 as lineage‐specific identifiers [28, 29, 30, 31]. Furthermore, RNF187 exhibits species‐divergent substrate specificity; in mouse spermatogonia (GC‐1) and spermatocytes (GC‐2), RNF187 regulates proliferation via ubiquitination of KRT36/KRT84 and histone H3 [32, 33], whereas our study reveals its human‐specific targeting of WDR77 to govern SSC maintenance through epigenetic derepression of early growth response protein 1 (EGR1). These disparities underscore the necessity of direct human SSC investigations to bridge mechanistic insights from mouse models to clinical applications, particularly for developing therapies targeting NOA caused by SSCs dysfunction.

## 2 Materials and Methods

### 2.1 Clinical Sample Acquisition

Testicular specimens were obtained from a male donor whose partner was enrolled in an in vitro fertilisation (IVF) programme at Suzhou Municipal Hospital. The tissues underwent sequential PBS washes followed by fixation in 4% paraformaldehyde. This study protocol was approved by the Ethics Committee of Suzhou Municipal Hospital (Approval: 2023005), with written informed consent secured from the participant in strict adherence to the ethical principles of the Declaration of Helsinki.

### 2.2 Cell Culture and Treatment

The human SSC line was kindly provided by Professor Zuping He at Hunan Normal University (China) [34, 35], which was cultured in Dulbecco's Modified Eagle Medium and nutrition Mixture F‐12 (DMEM/F‐12; Gibco, USA), supplemented with 10% fetal bovine serum (FBS; ScienCell, USA) and 1% penicillin–streptomycin solution (NCM Biotech, China). The culture medium was replaced every day. The culture dishes were kept in a humidified incubator at 37°C with a CO_2_ content of 5%.

The HEK293 cells utilised in this study were sourced from the American Type Culture Collection. Cells were cultured in DMEM (Gibco) supplemented with 10% fetal bovine serum (Gibco) under standard conditions (37°C, 5% CO_2_).

Rigorous mycoplasma screening was performed monthly and before critical experiments using MycoBlue Mycoplasma Detector (Vazyme, D101‐02), consistently yielding negative results.

### 2.3 Cell Transfection and Plasmids

For human SSCs, upon reaching the cell confluence of 60%–70%, we perform the cell transfection of small interfering RNA (siRNA) and overexpression plasmids. The siRNAs used in this study were obtained from Genepharma (Shanghai, China) and transfected using Lipofectamine iMAX (Invitrogen, USA) according to the manufacturer's instructions. All plasmids utilised in this experiment were obtained from Sangon Biotech Inc. (Shanghai, China) and were transfected following the protocols outlined for the X‐treme GENE HP DNA Transfection Reagent (Roche, Switzerland). After a transfection period of 6 h, the medium was replaced with complete medium. For all transfection procedures, the concentrations of plasmids and siRNAs used were 1 μg/mL and 50 nM, respectively. Cells were harvested 48 h post‐transfection for subsequent experiments.

For HEK293 cells, plasmids were introduced using Lipofectamine 2000 (Thermo Fisher) at a 3:1 reagent‐to‐DNA ratio, following the manufacturer's protocol, with cells harvested 48 h post‐transfection.

The specific target sequences of the siRNA are detailed in Table S1. Relevant information regarding the plasmids is provided in Table S2.

### 2.4 Quantitative Real‐Time Polymerase Chain Reaction (RT‐qPCR)

The RNA samples were extracted using Total RNA Extraction Reagent (Vazyme, Nanjing, China). The RNA was subsequently reverse transcribed into cDNA using a Prime Script Reverse Transcription Kit (Vazyme). The RT‐qPCR was performed using Taq Pro Universal SYBR qPCR Master Mix (Vazyme) and relative mRNA expression was detected with the Applied Biosystems 7500 real‐time PCR system. Gene expression levels were normalised to 18S rRNA. The primers used are provided in Table S3.

### 2.5 CCK‐8 Assay and Clonal Formation Experiment

For the CCK‐8 assay, 2 × 10^3^ transfected cells were seeded into 96‐well plates per well. Cell viability was evaluated every 24 h using the Cell Counting Kit‐8 (Beyotime, Nantong, China), as previously described [36, 37]. The absorbance of each well was measured using a microplate reader (Bio‐Rad Model 680, USA) at an optical density of 450 nm. For the colony formation assay, 1 × 10^3^ transfected cells were inoculated into six‐well plates. After 2 weeks of culturing, the proliferative capacity was observed by the number of stained colonies with 0.1% crystal violet (Beyotime).

### 2.6 Cell Migration Assays

The migration chamber used has a filter membrane with an 8 μm pore size (Corning, Corning, New York, USA) and is placed in a 24‐well plate. In the inner chamber, 300 μL of serum‐free medium and 4.5 × 10^4^ cells were added, while 700 μL of complete medium was added to the outer chamber. After 48 h of culturing, the cells that migrated to the outer wall of the chamber were fixed with methanol for 15 min and then stained with crystal violet for 15 min. After drying, random fields of view were selected under a microscope (Axioskop 2 Plus; Carl Zeiss, Oberkochen, Germany) for photography and cell counting for statistical analysis.

### 2.7 Immunoblotting

Immunoblotting was conducted as previously described [33]. In brief, the cell samples were lysed using radioimmunoprecipitation assay (RIPA, Beyotime) containing 1% protease inhibitor phenylmethylsulfonyl fluoride (PMSF, Beyotime). After high‐speed centrifugation, the supernatant was collected as the protein product. This product was then denatured in a metal bath at 100°C for 10 min following the addition of 5× sodium dodecyl sulfate (SDS). Primary antibodies and horseradish peroxidase‐conjugated secondary antibodies were incubated on the membrane. Finally, an Image‐Pro Plus (Media Cybernetics, USA) was used to measure the band signals detected by the BeyoECL Plus kit (Beyotime). Relevant information about the antibodies used in this study is provided in Table S4.

### 2.8 Immunoprecipitation (IP) and Liquid Chromatography–Mass Spectrometry (LC–MS/MS)

For endogenous IP, 25 μL of protein A/G beads (Thermo Scientific) were added to the lysate to remove non‐specific proteins that the magnetic beads could bind. The mixture was rotationally mixed at 4°C for 3 h. Subsequently, 5 μg of target antibody was added to the supernatant, mixed, and incubated overnight at 4°C. IgG was used as a negative control. After 12–14 h of incubation, 50 μL of protein A/G beads were added, and the mixture was incubated at 4°C for 3 h. The beads were then washed three times with RIPA lysis buffer containing 1% protease inhibitor. The beads were then resuspended in 50 μL of 1× SDS buffer and denatured by heating in a metal bath at 100°C for 10 min.

For exogenous IP, the procedures were applied to cells pre‐transfected with the indicated plasmids. Myc‐beads (AlpalifeBio, China) were added to the supernatant, mixed, and incubated at 4°C for 12–14 h. After removing the supernatant, the beads were washed three times with RIPA lysis buffer containing 1% protease inhibitor. Subsequent experimental steps were similar to the endogenous IP assay. The final IP product was analysed by immunoblotting or liquid chromatography–mass spectrometry tandem mass spectrometry (LC–MS/MS) [38, 39]. Following immunoprecipitation, proteins were eluted, reduced with 10 mM DTT, alkylated with 55 mM iodoacetamide, and digested overnight with sequencing‐grade trypsin (Promega). Desalted peptides were separated on a nanoElute UHPLC system (Thermo Scientific) using a 25‐cm Aurora C18 column with a 60 min gradient (2%–22% acetonitrile, 0.1% formic acid, 300 nL/min). Mass spectrometry was performed on a timsTOF Pro 2 (Bruker) in DDA‐PASEF mode, with MS1 (100–1700 m/z) and MS2 (intensity threshold 2500, dynamic exclusion 30 s). Raw data were processed via MaxQuant (v2.2.0) against the UniProt human database, allowing two missed cleavages, 20 ppm/0.05 Da mass tolerances.

### 2.9 Protein Half‐Life Assays

The cells were transfected with siRNA or plasmid for 48 h. Protein synthesis was inhibited by the addition of cycloheximide (CHX, 100 μg/mL) for 0 , 4 and 8 h before transfected cells were collected. Subsequently, the expression of WDR77 in total cellular proteins was detected by immunoblotting.

### 2.10 In Vitro Ubiquitination Assays

The in vitro ubiquitination assay was conducted with a ubiquitination kit (Enzo, Farmingdale, NY, USA) according to the manufacturer's instructions. Briefly, Flag‐tagged RNF187^WT^ and RNF187^MUT^ immunoprecipitates were purified using Flag beads from human SSCs and then mixed with a 50‐μL in vitro ubiquitination reaction system. The ubiquitination assay system consisted of E1 activating enzyme (100 nM), ubiquitin (2.5 μM), E2 conjugating enzyme (2.5 μM), Mg^2+^‐ATP (5 mM), dithiothreitol (1 mM) and 5 μg His‐tagged WDR77 recombinant protein (Sangon Biotech Inc., Shanghai, China). The mixture was incubated at 37°C for 8 h. The final reaction product was assessed for ubiquitination level by immunoblotting.

### 2.11 RNA‐Seq

The RNA extracted from the cells was initially subjected to quality control. mRNAs with poly(A) tails were enriched using oligo(dT) magnetic beads. These mRNAs were then randomly fragmented using divalent cations in a specified buffer, and library construction was performed following the NEB standard library construction protocol. Preliminary quantification was conducted using a Qubit 2.0 Fluorometer, followed by accurate determination of the libraries' effective concentration via RT‐qPCR. After successful library validation, the different libraries were pooled based on their effective concentrations and the required data volume for downstream Illumina sequencing. Differentially expressed genes were defined based on a fold change > 1.5 and a false discovery rate (FDR) < 0.05.

### 2.12 Chromatin Immunoprecipitation (ChIP)

ChIP experiments were performed with the EZ‐ChIP kit (Millipore, Billerica, MA, United States) as previously describe [40]. In general, cells were collected and then cross‐linked with 1% formaldehyde, followed by using a Branson Sonicator 250 to shear into ∼500‐bp fragments, and then the chromatin DNA–protein complex was reacted with the indicated antibodies (Table S4). Approximately 10% of the starting material was used as input. Immunoprecipitated DNA was analysed by real‐time PCR. The primers used are provided in Table S3.

### 2.13 Luciferase Reporter Assay

Empty vector and *EGR1*‐promoter were sub‐cloned into the pGL6 luciferase vector to construct the pGL6‐EV and pGL6‐*EGR1*‐promoter. Then, plasmids were transfected into human SSCs along with various small interfering RNAs or plasmids. Renilla luciferase vector was used as an internal control reporter. The luciferase activity was detected via Luciferase Reporter Assay System [40].

### 2.14 Statistical Analyses

Statistical analyses were performed using GraphPad Prism 7.0 (GraphPad Software, La Jolla, CA, USA). Unpaired Student's t‐test was used to assess the significance of differences between two groups; one‐way ANOVA was used to assess the significance of differences between more than two groups. *p* < 0.05 was considered statistically significant.

## 3 Result

### 3.1 RNF187 Is Indispensable for the Proliferation and Migration of Human SSCs

**FIGURE 1:**
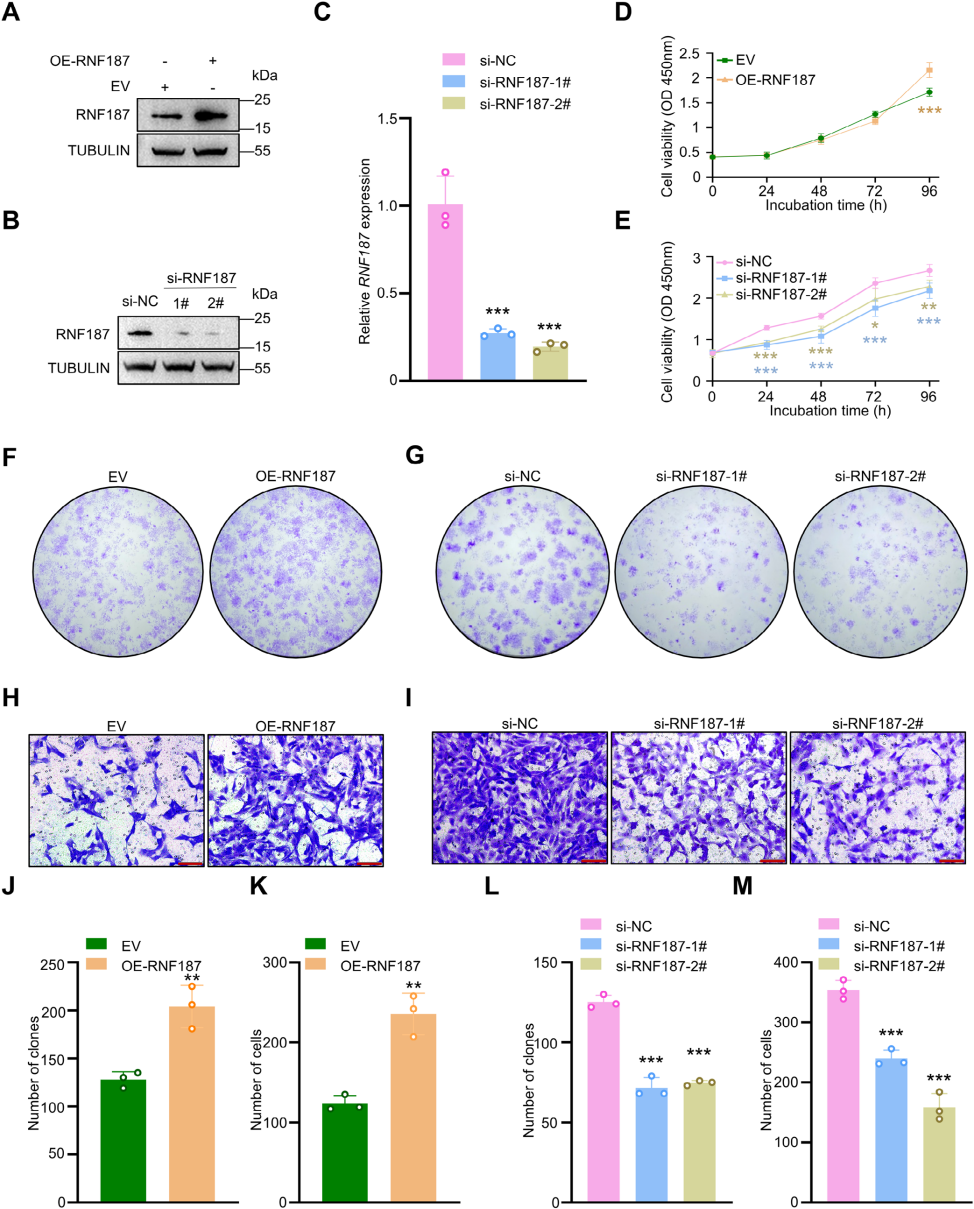
RNF187 increases the proliferation and migration of SSCs. (A, B) Immunoblotting was used to determine the protein level of RNF187 in SSCs. Similar results were observed in three independent experiments. (C) Relative expression of *RNF187* were measured by RT‐qPCR in SSCs transfected with si‐NC, si‐*RNF187*‐1#, or si‐*RNF187*‐2# (*n* = 3). (D) CCK‐8 assays were conducted to assess the viability of SSCs overexpressing *RNF187* (*n* = 6). (E) CCK‐8 assays were conducted to assess the viability of *RNF187*‐knockdown SSCs (*n* = 6). (F) Colony formation assays were performed to assess the proliferative ability of SSCs overexpressing *RNF187* (*n* = 3). (G) Colony formation assays were performed to assess the proliferative ability of *RNF187*‐knockdown SSCs (*n* = 3). (H) Transwell assays were carried out to evaluate the migration of SSCs overexpressing *RNF187* (*n* = 3). Scale bar:100 μm. (I) Transwell assays were carried out to evaluate the migration of SSCs *RNF187*‐knockdown SSCs (*n* = 3). Scale bar: 100 μm. (J and K) Quantitative analysis of the results in (F and H). (L and M) Quantitative analysis of the results in (G and I). Data are shown as means ± SD; Unpaired Student's *t*‐test (D, J, K); One‐way ANOVA (E, L, M); *p < 0.05, **p < 0.01, ***p < 0.001.

SSCs uniquely possess the capability of self‐renewal, a process essential for the regulation of spermatogenesis [41]. We observed co‐localization of RNF187 with PLZF, a marker of SSCs, in human testicular tissues, suggesting that RNF187 plays a crucial role in maintaining SSC functionality (Figure S1). Utilising a human SSC cell line, we further investigated how RNF187 regulates the proliferation and migration of human SSCs. The efficiency of knockdown or overexpression for *RNF187* was confirmed via immunoblotting or RT‐PCR (Figure 1A–C). CCK‐8 assays demonstrated that overexpression or knockdown of *RNF187* significantly enhanced and reduced cell proliferation levels, respectively (Figure 1D,E). Additionally, overexpression or knockdown of *RNF187* respectively promotes and suppresses SSCs proliferation in 2D monolayer cultures (Figure 1F,G,J,L). The movement of proliferating SSCs into stem cell niches created by the division of Sertoli cells is crucial for the process of spermatogenesis [42]. We subsequently performed a transwell assay across a Matrigel membrane and observed that overexpression or knockdown of *RNF187* respectively enhanced and diminished the migration ability of SSCs through the gel matrix into the adjacent chamber (Figure 1H,I,K,M). In conclusion, RNF187 is essential for sustaining the proliferation and migration of human SSCs.

### 3.2 WDR77 Is a Specific Substrate of RNF187 and Is Degraded Through UPS in Human SSCs

**FIGURE 2:**
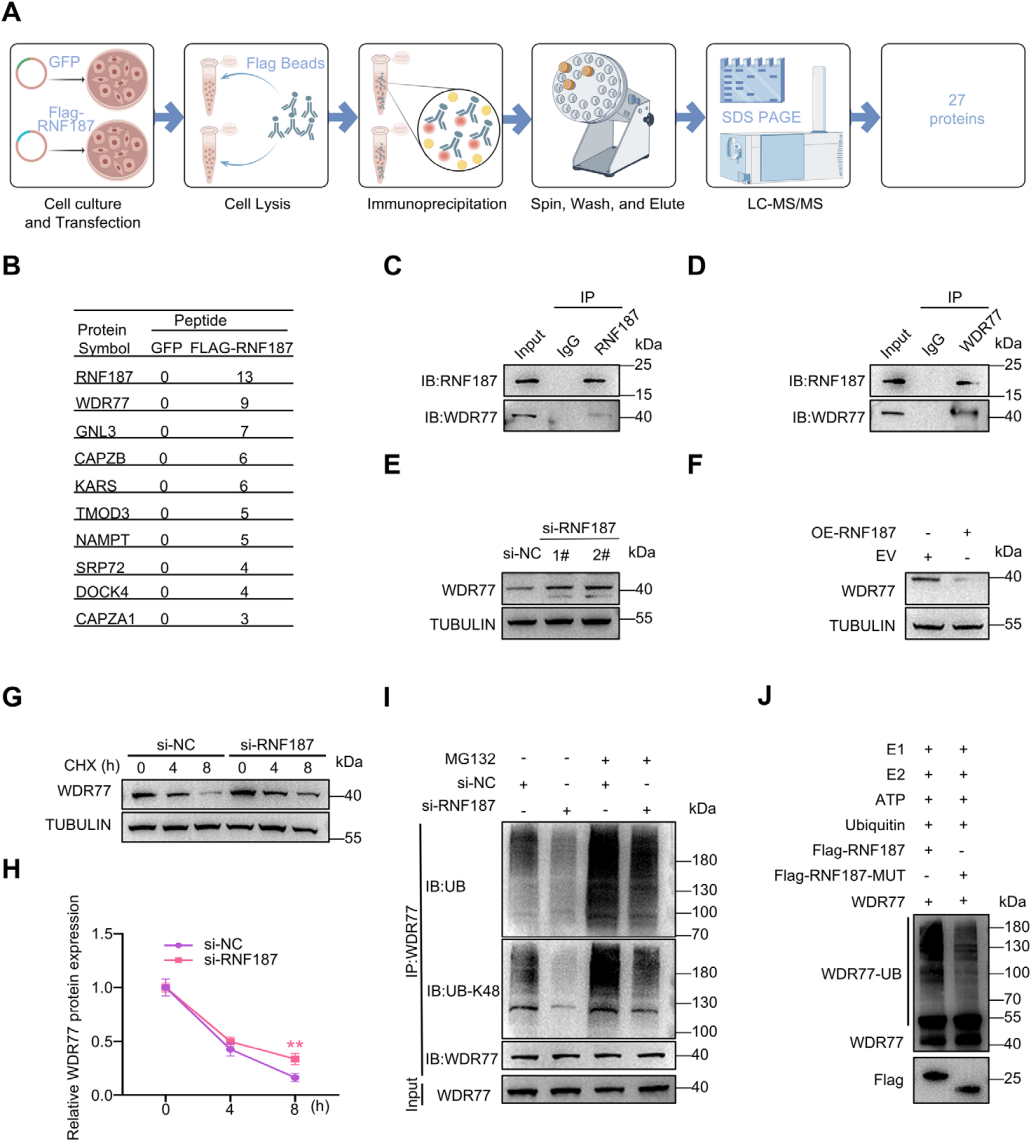
RNF187 interacts with WDR77. (A) Procedures used to identify RNF187‐interacting proteins in SSCs: Total proteins were extracted after transfection, followed by immunoprecipitation with Flag‐Beads, SDS‐PAGE, in‐gel digestion and LC‐MC/MS. (B)Proteins ranked in the top 10 by peptide number in the mass spectrometry results. (C, D) Immunoprecipitation and immunoblotting showed the endogenous interactions between RNF187 and WDR77 in human SSCs. Similar results were observed in three independent experiments. (E, F) Immunoblotting using anti‐WDR77 and anti‐tubulin antibodies after knockdown and overexpression of *RNF187*‐treated cells for 48 h. Similar results were observed in three independent experiments. (G) Immunoblotting analysis of WDR77 expression in SSCs transfected with si‐*RNF87* and treatment with cycloheximide (CHX) for 0, 4 and 8 h. Similar results were observed in three independent experiments. (H) Quantification of half‐life of WDR77 protein in (G), *n* = 3. (I) After transient transfection of si‐NC and si‐*RNF187*, cells were treated with 20 μM MG132 for 6 h. WDR77 immunoprecipitates in SSCs were then purified by WDR77 antibody, and WDR77 polyubiquitination levels were detected by immunoblotting using anti‐UB and anti‐UB‐K48 antibodies. Similar results were observed in three independent experiments. (J) In vitro ubiquitination of WDR77 by Flag‐RNF187 immune complexes. Flag‐RNF187‐MUT was used as the negative control. Both Flag‐RNF187 and Flag‐RNF187‐MUT immunoprecipitates were obtained from human SSC lysates. Similar results were observed in three independent experiments. Data are shown as means ± SD; Unpaired Student's *t*‐test; **p < 0.01.

To investigate the molecular mechanisms by which RNF187 regulates human SSCs proliferation and migration, we further explored the interacting proteins of RNF187. Human SSCs were transfected with GFP and Flag‐RNF187 plasmids, and immunoprecipitation was performed using an antibody against the exogenous Flag tag on the lysates of transfected SSCs. The isolated Flag‐RNF187 was then analysed by liquid chromatography–tandem mass spectrometry (LC–MS/MS) (Figure 2A). Through this process, we successfully identified 27 candidate proteins. Among these proteins, WDR77 was selected for further analysis due to its peptide count being only lower than RNF187. The genes of the top 10 identified proteins are listed in Figure 2B, with the complete dataset provided in Dataset S1. Immunoprecipitation using SSCs lysates also confirmed the endogenous interactions between WDR77 and RNF187 (Figure 2C,D).

We then examined whether the interactions between RNF187 and WDR77 influence WDR77 stability. Immunoblotting results indicated that knockdown or overexpression of *RNF187* in human SSCs respectively reduced or enhanced WDR77 expression (Figure 2E,F). To investigate the mechanism of the obvious accumulation of WDR77 induced by *RNF187* knockdown, we performed a cycloheximide (CHX) chase assay to measure the half‐life of WDR77 in control and *RNF187* knockdown SSCs, and the results showed delayed degradation of WDR77 in *RNF187* knockdown SSCs (Figure 2G,H). The E3 ubiquitin ligase RNF187 mediates substrate protein degradation via the ubiquitin‐proteasome pathway (UPS) [32]. We therefore treated SSCs with MG132, a pivotal inhibitor of the UPS. Immunoblotting results showed that in *RNF187* knockdown SSCs, treatment with MG132 significantly reduced the ubiquitination levels of endogenous WDR77 compared to control SSCs (Figure 2I). An in vitro ubiquitination assay also demonstrated that wild‐type RNF187, but not the inactive mutant RNF187 [33], could efficiently catalyse the formation of polyubiquitin chains on WDR77 (Figure 2J). Therefore, RNF187 mediates the degradation of the substrate WDR77 through polyubiquitination.

### 3.3 RNF187 Mediates the Ubiquitination of WDR77 at the K118 Site

**FIGURE 3:**
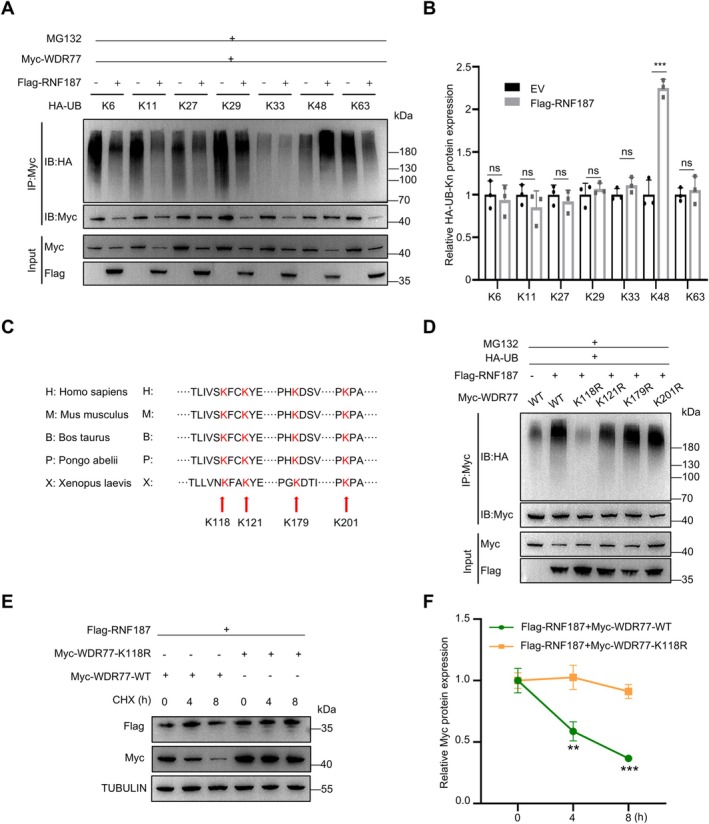
RNF187 mediates K48‐linked ubiquitination of WDR77 at the K118 site. (A) Co‐transfected HA‐UB (WT, K6, K11, K27, K29, K33, K48, K63), Myc‐WDR77, and Flag‐RNF187 plasmids in HEK293 cells were subjected to Co‐IP assay, and ubiquitination level of WDR77 was detected by immunoblotting. MG132 was used at a concentration of 20 μM. Similar results were observed in three independent experiments. (B) Quantification of ubiquitination level of WDR77 in (A). Similar results were observed in three independent experiments. (C) Predicted ubiquitination sites of WDR77 in different species. (D) Screening for potential ubiquitination sites of WDR77 in HEK293T cells. The polyubiquitination of WDR77 in response to RNF187 overexpression was assessed in cells transfected with the indicated plasmids and treated with MG132 (20 μM). Similar results were observed in three independent experiments. (E) HEK293 cells transfected with Myc‐WDR77 and Myc‐WDR77^K118R^ were treated with CHX at 0, 4 and 8 h before harvest, and Myc‐tagged protein were detected by immunoblotting. Similar results were observed in three independent experiments. (F) Quantification of Myc‐tagged protein expression levels in (E). Data are shown as means ± SD; Unpaired Student's *t*‐test; **p < 0.01, ***p < 0.001, ns: Not significant.

Ubiquitin contains seven internal lysine residues (K6, K11, K27, K29, K33, K48 and K63), which serve as acceptor sites for UB conjugation and the formation of distinct ubiquitin chains [43]. Screening for mutated forms of ubiquitin to identify potential lysine ubiquitination sites revealed that only UB‐K48 (UB with the intact Lys48 residue), not K6, K11, K27, K29, K33 or K63, could be conjugated to WDR77 by RNF187 (Figure 3A,B). We utilised GPS‐Uber [44], a tool for predicting ubiquitin‐protein ligase enzyme‐substrate relationships, to identify potential lysine residues. GPS‐Uber predicted four evolutionarily conserved ubiquitylation sites K118, K121, K179 and K201 in WDR77. We then mutated the lysine residues at these ubiquitination sites on human WDR77 to arginine and overexpressed them together with HA‐ubiquitin and Flag‐RNF187 in HEK293 cells. After treatment with MG132, immunoprecipitation of SSCs lysates revealed that the ubiquitination level of WDR77^K118R^, but not other WDR77 mutants, was significantly reduced compared to the positive control (Figure 3D). This suggests that the K118 site is a key ubiquitination site of WDR77 targeted by RNF187. To further confirm that RNF187 mediates the degradation of WDR77 through the UPS by ubiquitinating the K118 site, we treated Flag‐RNF187‐transfected HEK293 cells with CHX and compared the degradation of Myc‐WDR77^WT^ and Myc‐WDR77^K118R^. The results showed delayed degradation of WDR77^K118R^ in Flag‐RNF187‐transfected HEK293 cells (Figure 3E,F).

### 3.4 Ubiquitination of WDR77 Is Required for the Proliferation and Migration of SSCs

**FIGURE 4:**
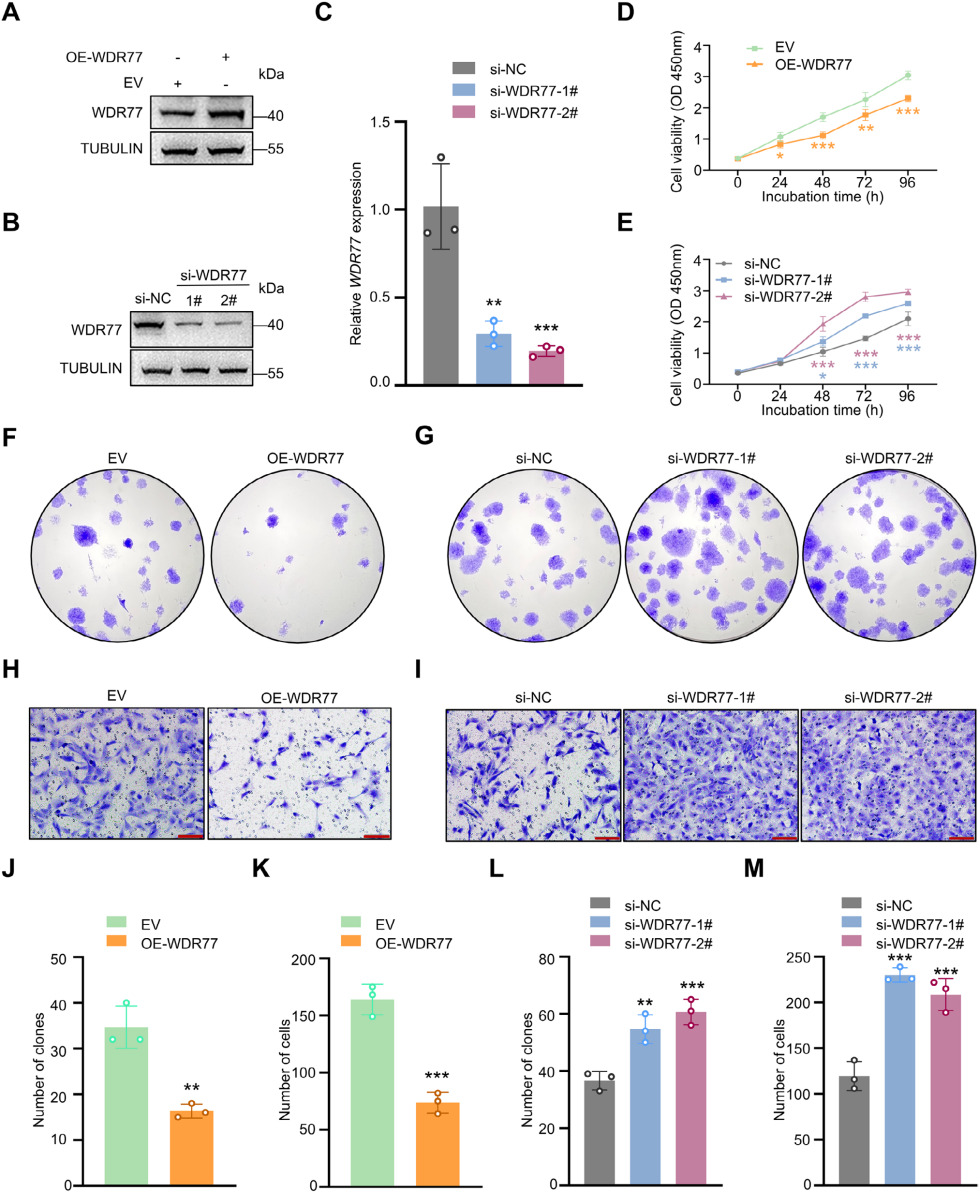
WDR77 inhibits proliferation and migration of human SSCs. (A, B) Immunoblotting was used to determine the protein level of WDR77 in SSCs. Similar results were observed in three independent experiments. (C) Relative expression levels of WDR77 were measured by RT‐qPCR in SSCs transfected with si‐NC, si‐*WDR77*‐1#, or si‐*WDR77*‐2# (*n* = 3). (D) CCK‐8 assays were conducted to assess the viability of SSCs overexpressing WDR77 (*n* = 6). (E) CCK‐8 assays were conducted to assess the viability of *WDR77*‐knockdown SSCs (*n* = 6). (F) Colony formation assays were performed to assess the proliferative ability of SSCs overexpressing *WDR77* (*n* = 3). (G) Colony formation assays were performed to assess the proliferative ability of *WDR77*‐knockdown SSCs (*n* = 3). (H) Transwell assays were carried out to evaluate the migration of SSCs overexpressing *WDR77* (*n* = 3). Scale bar: 100 μm. (I) Transwell assays were carried out to evaluate the migration of *WDR77*‐knockdown SSCs (*n* = 3). Scale bar: 100 μm. (J, K) Quantitative analysis of the results in (F and H). (L, M) Quantitative analysis of the results in (G and I). Data are shown as means ± SD; Unpaired Student's *t*‐test (D, J, K); One‐way ANOVA (E, L, M); *p < 0.05, **p < 0.01, ***p < 0.001.

Given that the ubiquitination and deubiquitination of WDR77 respectively promote and inhibit its degradation via the UPS. We simulated the ubiquitinated and deubiquitinated states of WDR77 by knocking down and overexpressing *WDR77*, respectively, to study how RNF187‐mediated ubiquitination of WDR77 affects SSCs proliferation and migration. The efficiency of knockdown or overexpression for *WDR77* was confirmed via immunoblotting or RT‐PCR (Figure 4A–C). Overexpressing or knocking down *WDR77* in SSCs significantly impaired or enhanced their proliferation and migration, respectively (Figure 4D–M).

### 3.5 The RNF187‐WDR77‐EGR1 Pathway Is Essential for SSC Proliferation and Migration

**FIGURE 5:**
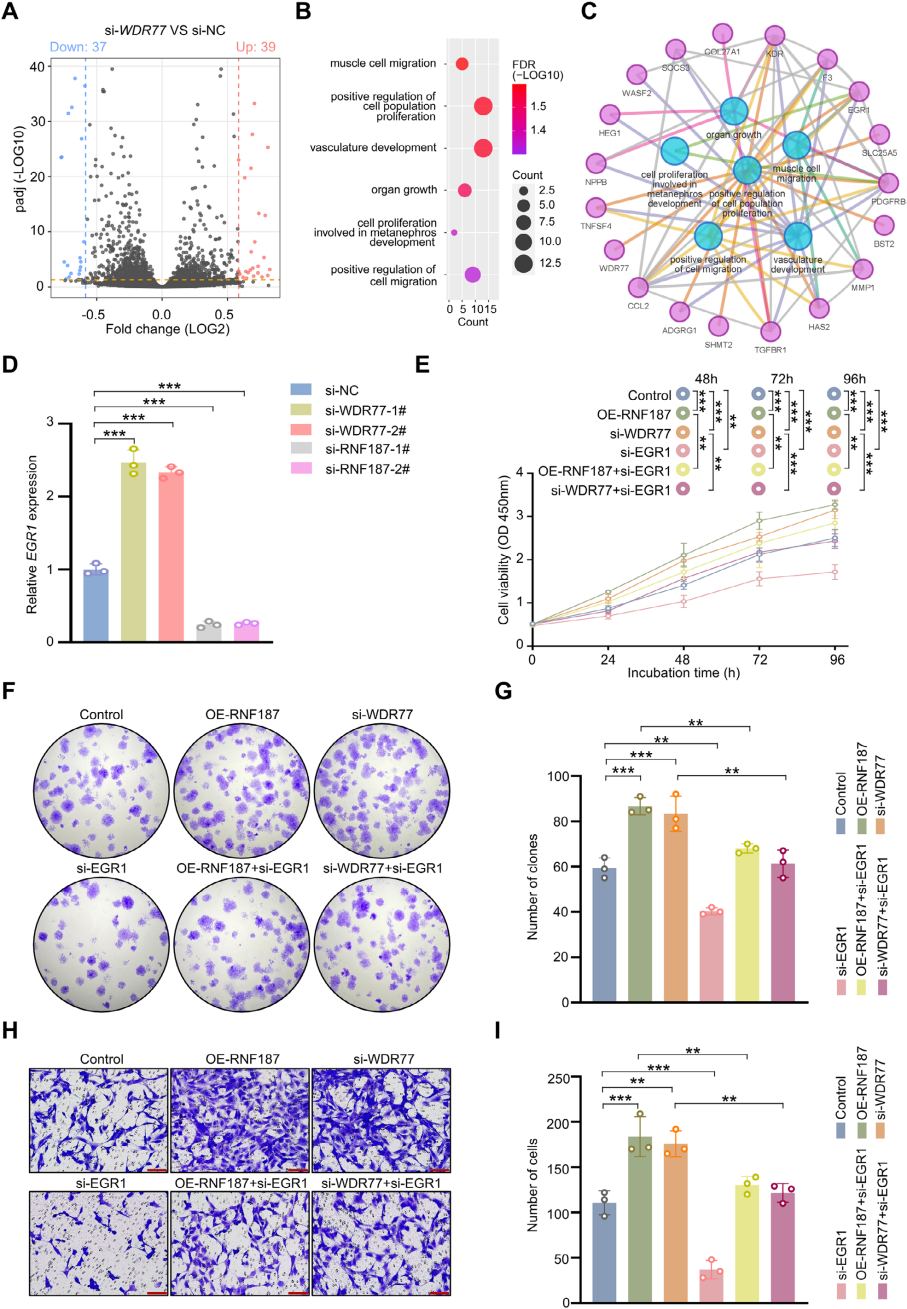
EGR1 is a downstream target of WDR77 and can rescue the proliferation and migration of SSCs induced by *RNF187* overexpression and *WDR77* knockdown. (A) Three biological replicates of si‐*WDR77* and si‐NC SSCs were subjected to RNA‐seq. This volcano plot shows the differences in gene expression between the si‐*WDR77*‐treated group and the si‐NC control group, blue points indicate significantly downregulated genes, red points indicate significantly upregulated genes, and grey points indicate non‐significant genes. The dotted line indicates the significance threshold. (B) Differentially expressed genes obtained by RNA‐seq in Gene Ontology (GO) enrichment analysis. (C) Enrichment and correlation of differentially expressed genes obtained by RNA‐seq in multiple important biological processes. (D) RT‐qPCR analysis was used to determine *EGR1* gene expression in SSCs transfected with si‐NC, si‐*RNF187*, or si‐*WDR77* (*n* = 3). (E‐I) After transfection of SSC cells with si‐NC + EV (Control), OE‐*RNF187*, si‐*WDR77*, si‐*EGR1*, si‐*EGR1* + OE‐*RNF187*, and si‐*EGR1* + si‐*WDR77*, respectively, EV: Empty vector. (E) Cell viability was assayed by CCK8 (*n* = 6). (F, G) Cell proliferation capacity was determined by colony formation assay (*n* = 3). (H, I) Migration ability was studied by transwell assay (*n* = 3). Scale bar: 100 μm. Data are shown as means ± SD; One‐way ANOVA; **p < 0.01, ***p < 0.001.

To explore how RNF187‐mediated ubiquitination of WDR77 influences the proliferation and migration of SSCs. We performed whole transcriptome analysis using RNA‐seq to gain insight into the consequences of *WDR77* knockdown in SSCs. The volcano plots showed that, compared to the negative control, the SSCs with *WDR77* knockdown featured 76 significantly differentially expressed genes (DEGs), with 39 upregulated and 37 downregulated (Figure 5A, Dataset S2). GO enrichment analysis and pathway network mapping of the 76 DEGs suggest that these genes are primarily associated with cell proliferation and migration, which was consistent with our observations that knockdowns of *WDR77* in SSCs result in reduced cell proliferation and migration (Figure 5B,C). Notably, *EGR1* was upregulated in SSCs with *WDR77* knockdown. RT‐PCR results indicated that the expression of WDR77 and RNF187 was negatively and positively correlated with the expression of EGR1, respectively (Figure 5D).

Previous studies have demonstrated that EGR1 is essential for the proliferation of human SSCs [45]. We have validated the knockdown efficiency of EGR1 in human SSCs through RT‐PCR; siRNA‐mediated knockdown of *EGR1* significantly reduces *EGR1* mRNA levels in human SSCs (Figure S2). Our findings further confirm that the knockdown of *EGR1* in human SSCs impairs both their proliferation and migration (Figure 5E–I). Notably, the overexpression of *RNF187* and the knockdown of *WDR77* in *EGR1* knockdown SSCs partially rescued their proliferation and migration (Figure 5E–I). These results suggest that the RNF187‐WDR77‐EGR1 pathway plays a crucial role in regulating SSCs proliferation and migration.

### 3.6 WDR77/PRMT5 Inhibits the Transcription and Expression of *EGR1* by Catalysing H4R3me2

**FIGURE 6:**
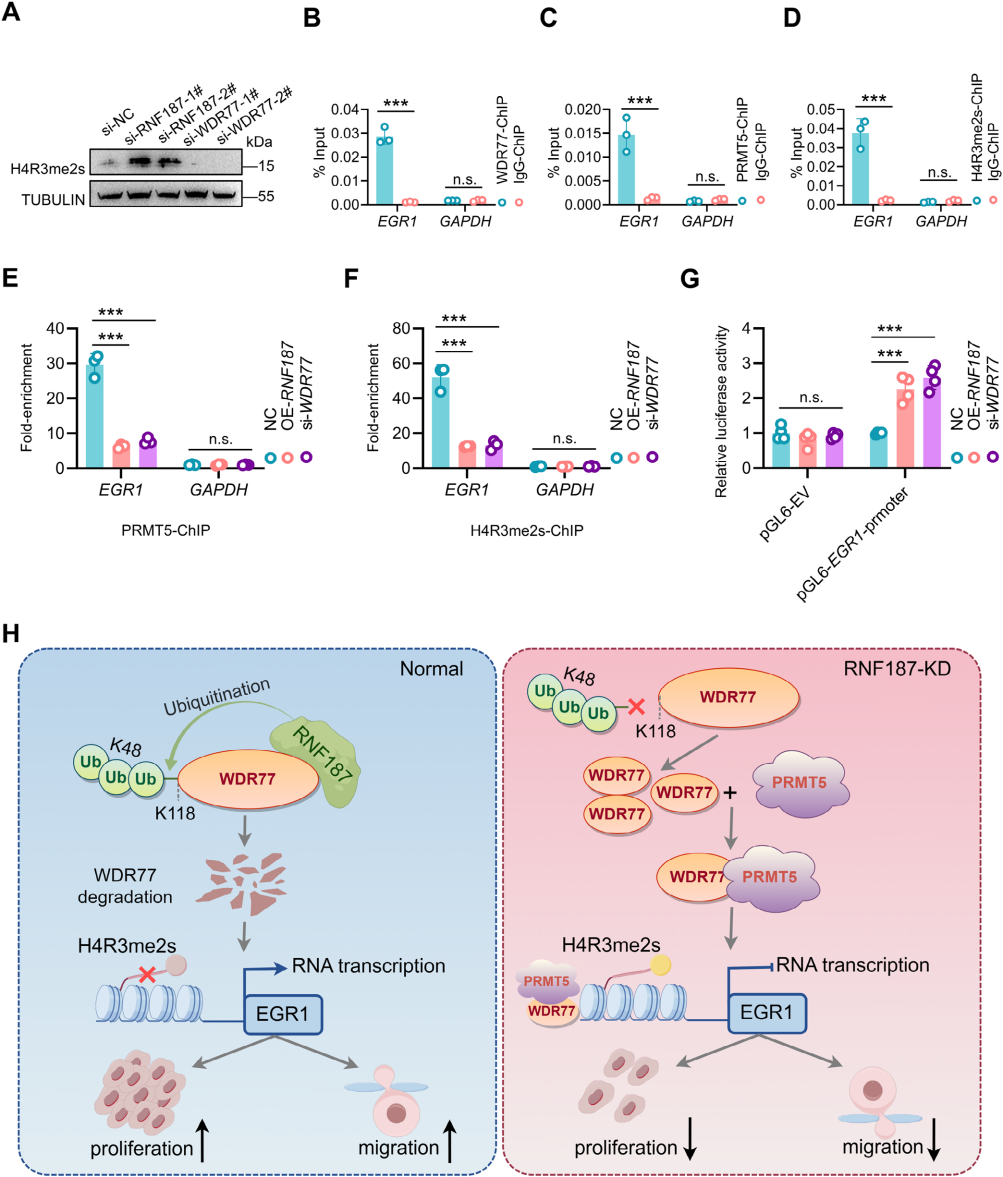
RNF187 eliminates transcriptional repression of EGR1 induced by WDR77/PRMT5. (A) Immunoblotting analysis of H4R3me2s in human SSCs transfected with si‐NC, si‐*RNF187*, si‐*WDR77*. Similar results were observed in three independent experiments. (B‐D) ChIP‐qPCR of WDR77‐related (B), PRMT5‐related (C) and H4R3me2s‐related (D) DNA sequences from the promoter region of *EGR1* in human SSCs (*n* = 3). The *GAPDH* gene was used as a negative control. (E, F) ChIP‐qPCR of PRMT5‐associated (E) and H4R3me2s‐associated (F) DNA sequences from the promoter region of *EGR1* in OE‐*RNF187*‐treated or si‐*WDR77*‐treated human SSCs (*n* = 3). (G) Measurement of luciferase activity in human SSCs transfected with the indicated vectors and siRNA (*n* = 4). (H) Scheme of RNF187 regulation of human SSCs proliferation and migration. Data are shown as means ± SD; Unpaired Student's *t*‐test (B, C, D); One‐way ANOVA (E, F, G); ***p < 0.001, ns: Not significant.

We further investigated how WDR77 regulates EGR1 expression. WDR77 interacts with protein arginine methyltransferase 5 (PRMT5) to form a histone methyltransferase complex that catalyses the dimethylation of H4R3 (H4R3me2), thereby promoting cancer cell proliferation and migration [45]. Our findings also confirm that knockdown of *WDR77* in SSCs leads to a reduction in H4R3me2 levels, whereas knockdown of *RNF187* in SSCs results in an increase in H4R3me2s expression (Figure 6A). Then, we performed ChIP experiments with anti‐WDR77, PRMT5, and H4R3me2s antibodies. As expected, these proteins showed significant binding to the promoter region of *EGR1*, but not to the *GAPDH* (Figure 6B–D). Subsequent ChIP experiments revealed that overexpression of *RNF187* and inhibition of *WDR77* significantly reduced the binding levels of PRMT5 and H4R3me2s to the promoter region of *EGR1* (Figure 6B–F). From luciferase reporter assays using pGL6‐basic constructs, in which *EGR1*‐promoter harbours the pairing sequence, we found that upregulation of *RNF187* or downregulation of *WDR77* significantly enhanced the luciferase activity in human SSCs transfected with the *EGR1*‐promoter reporter gene construct (Figure 6G). In summary, these data suggest that RNF187 mediates the ubiquitination of WDR77, reduces H4R3me2s catalysed by the WDR77/PRMT5 complex, and alleviates the transcriptional repression of *EGR1*. The increased expression of EGR1 subsequently promotes the proliferation and migration of human SSCs (Figure 6H).

## 4 Discussion

The seminiferous epithelium is one of the most intricate epithelial structures, with SSCs located at its base playing a pivotal role in spermatogenesis through self‐renewal and differentiation [46]. Human and mouse SSCs show significant differences. Studies have demonstrated that POU5F1 is not expressed in human SSCs, whereas it is specifically expressed in mouse SSCs [29, 47]. Additionally, UTF1 and FGFR3 serve as specific markers for human SSCs and are crucial for the identification and isolation of human SSCs. However, these markers are not expressed in mouse SSCs [28, 30, 31]. These findings suggest that the molecular regulators and signalling pathways governing mouse SSCs may not be directly transferable to the study and application of human SSCs. Studies have demonstrated that FBXW7, a component of the Skp1‐Cullin‐F‐box‐type ubiquitin ligase complex, functions as a negative regulator of self‐renewal in mouse SSCs [48]. In contrast, the distinct expression pattern of FBXW7 in human male germ cells suggests that it plays a regulatory role during the later stages of spermatogenesis, rather than being involved in SSCs self‐renewal [49]. Thus, while previous studies conducted in transgenic mice have demonstrated that the absence of E3 ubiquitin ligases in SSCs disrupts normal spermatogenesis, the crucial ubiquitin ligases that govern the fate decisions of human SSCs have not yet been elucidated [49, 50]. In this study, we discovered that the E3 ubiquitin ligase RNF187 regulates the proliferation and migration of human SSCs through the WDR77/PRMT5‐H4R3me2s‐EGR1 signalling pathway, playing a crucial role in the fate determination of human SSCs (Figure 6H).

Previous studies have shown that the same protein can serve distinct functions at various stages of male germ cell development. In the early stages of spermatogenesis, PIWI interacts with eIF3F and ELAV1 to form the PIWI/eIF3F/ELAV1 translation activation complex, which plays a key role in activating mRNA translation [51]. In contrast, during the round spermatid stage, PIWI associates with the deadenylase CAF1 to form the PIWI/CAF1 degradation complex [52]. This complex triggers the large‐scale degradation and clearance of mRNA in the round spermatid cytoplasm, effectively preparing the cell for spermiogenesis. Inactivation of the E3 ubiquitin ligase HUWE1 in spermatogonia leads to the accumulation of the DNA damage response protein H2AX and subsequent apoptosis, thereby hindering the transition to pachytene spermatocytes [53]. However, HUWE1 inactivation in spermatocytes does not impact meiosis or spermiogenesis, indicating that HUWE1 functions differently at various stages of germ cell development. Our previous research demonstrated that RNF187 plays an indispensable role in the proliferation and migration of GC‐1 cells originating from spermatogonia and GC‐2 cells derived from spermatocytes. Notably, RNF187 regulates these processes in GC‐1 and GC‐2 cells by ubiquitinating KRT36/KRT84 and histone H3, respectively [32, 33]. In this study, we have further elucidated that RNF187 modulates the proliferation and migration of human SSCs via the ubiquitination of its downstream substrate WDR77. We attribute this difference to the fact that spermatogenesis is a highly regulated dynamic process involving mitotic proliferation, meiosis and cellular remodelling. This complex biological process necessitates that ubiquitin ligases recognise different substrates to facilitate protein turnover.

WDR77 was selected for in‐depth analysis primarily due to its interaction with PRMT5, a methyltransferase critical for histone modification that has been extensively implicated in tumour proliferation and migration. While other candidates, such as HSPA8 and HIST1H2BK, were identified in the IP‐MS screen, their roles in protein folding or chromatin organisation lack direct relevance to SSC‐specific regulation [54, 55]. Notably, mitochondrial proteins including ATP5F1 and COX4I1, as well as cytoskeletal regulators such as TUBA1B and TUBB2A, were excluded due to their broad roles in basic cellular functions rather than SSC fate determination [56, 57, 58, 59, 60]. Future studies will explore secondary candidates such as myeloid‐derived growth factor MYDGF and TNF receptor‐associated factor TRAF4, which may modulate stem cell regulatory pathways [61, 62].

Previous studies have demonstrated that WDR77‐PRMT5 catalyse symmetric dimethylation at H4R3 (H4R3me2s), thereby driving the proliferation and migration of cancer cells [63]. The deacetylase SIRT7 directly deacetylates WDR77, which disrupts the interaction between WDR77 and PRMT5 and subsequently inhibits the proliferation of human colon cancer HCT116 cells [45]. This finding suggests a positive correlation between WDR77 and cell proliferation. In the present study, we discovered that the RNF187‐mediated UPS pathway promotes the degradation of WDR77, thereby disrupting the WDR77‐PRMT5 interaction and reducing H4R3me2s levels. This disruption ultimately leads to the derepression of transcription of EGR1, a positive regulator of human SSCs proliferation [64]. Consequently, this derepression promotes the proliferation and migration of human SSCs. Our findings indicate a negative correlation between WDR77 and the proliferation of human SSCs, which contrasts with observations in tumour cells. We hypothesise that this discrepancy may be attributed to differences between germ cells and somatic cells. It has been previously reported that overexpression of TCF3 inhibits the proliferation of HCT116 colorectal cancer cells, whereas in human SSCs, TCF3 stimulates SSC proliferation and inhibits apoptosis by targeting podocalyxin‐like protein (PODXL) [65, 66].

This study establishes RNF187 as the first E3 ubiquitin ligase governing human SSCs proliferation and migration through lysine 48‐linked polyubiquitination of WDR77, a mechanism distinct from its role in mouse germ cells where it targets keratins (KRT36/KRT84) or histone H3 [32, 33]. We delineate a previously unrecognised RNF187‐WDR77‐EGR1 axis that bridges ubiquitin‐proteasome degradation with epigenetic regulation, demonstrating that RNF187‐mediated WDR77 turnover reduces PRMT5‐dependent H4R3me2s levels, thereby relieving transcriptional repression of EGR1. This pathway diverges fundamentally from oncogenic PRMT5/WDR77 signalling in cancer [22, 23], highlighting a unique regulatory paradigm in human SSCs. These insights advance the understanding of post‐translational control in human spermatogenesis and provide a molecular framework for targeting SSCs dysfunction in NOA.

## Author Contributions

**Haoyue Hu:** investigation, methodology, writing – original draft. **Xiaoxue Xi:** investigation, data curation. **Bing Jiang:** investigation, validation. **Kehan Wang:** methodology. **Tiantian Wu:** data curation. **Xia Chen:** data curation. **Yueshuai Guo:** methodology. **Tao Zhou:** methodology. **Xiaoyan Huang:** supervision. **Jun Yu:** conceptualisation, funding acquisition, project administration, supervision. **Tingting Gao:** conceptualisation, funding acquisition, project administration, supervision. **Yibo Wu:** conceptualisation, project administration, supervision. **Bo Zheng:** conceptualisation, funding acquisition, project administration, supervision, writing – review and editing.

## Ethics Statement

This study protocol was approved by the Ethics Committee of Suzhou Municipal Hospital (Approval: 2023005).

## Conflicts of Interest

The authors declare no conflicts of interest.

## Supporting information


Data S1.

Data S2.

Data S3.

## Data Availability

The mass spectrometry proteomics data have been deposited to the ProteomeXchange Consortium via the PRIDE partner repository with the dataset identifier PXD060599. The RNA‐Seq datasets are available in the ArrayExpress repository (accession number E‐MTAB‐14861).
