# Supplementary material for: RNF187 Facilitates Proliferation and Migration of Human Spermatogonial Stem Cells Through WDR77 Polyubiquitination
Source: Cell Prolif. 2025 Apr 8;58(10):e70042. doi: 10.1111/cpr.70042 (PMC12508693; doi:10.1111/cpr.70042)
Supplement: Supplementary file 3 — Data S3. [file CPR-58-e70042-s001.docx]

**Supplemental Materials**

**E3 Ubiquitin Ligase RNF187 Facilitates Proliferation and Migration of Human Spermatogonial Stem Cells through Lysine 48-Linked Polyubiquitination-Mediated Degradation of WDR77**

Haoyue Hu^1^, Xiaoxue Xi^2^, Bing Jiang^1^, Kehan Wang^2^, Tiantian Wu^2^, Xia Chen^3^, Yueshuai Guo^4^, Tao Zhou^5^, Xiaoyan Huang^4^, Jun Yu^6^, Tingting Gao^7^, Yibo Wu^1^, Bo Zheng^2^

Corresponding authors: Bo Zheng ([bozheng@njmu.edu.cn](mailto:bozheng@njmu.edu.cn)); Yibo Wu ([moliaty@aliyun.com](mailto:moliaty@aliyun.com)); Tingting Gao ([960182162@qq.com](mailto:960182162@qq.com)); Jun Yu ([yujun9117@ntu.edu.cn](mailto:yujun9117@ntu.edu.cn)).

**This file includes:**

Figure S1 to S2

Tables S1 to S4

Provided separately as Supplementary Dataset files

Dataset S1: RNF187 interactors identified in this study

Dataset S2: Differentially expressed genes identified between si-WDR77 and si-NC in this study


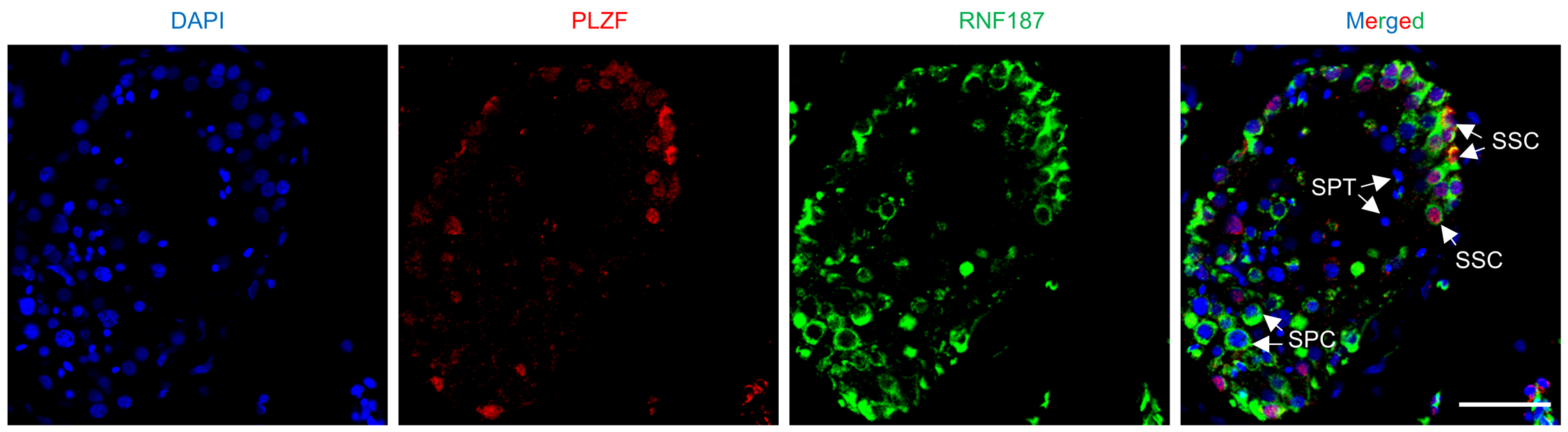


**Figure S1**. Co-immunostaining of RNF187 and PLZF (SSC marker) in human testis. RNF187 was partially co-expressed with PLZF in SSCs. In addition, RNF187 expression was also detected in spermatocytes. SSC: Spermatogonial stem cell; SPC: Spermatocyte; SPT: Spermatid. Scale bar: 50 μm.


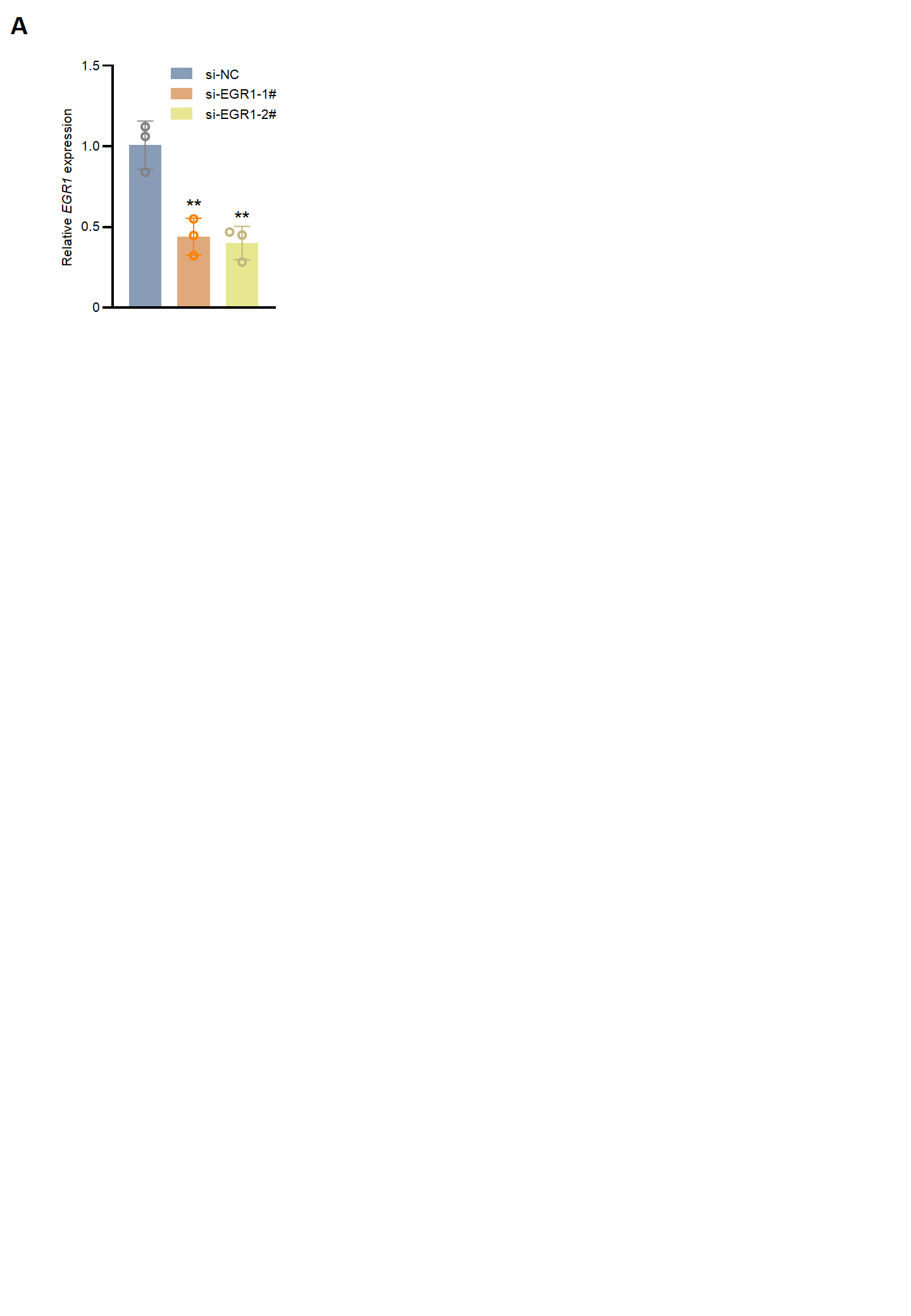


**Figure S2.** Relative expression levels of EGR1 were measured by RT-qPCR in SSCs transfected with si-NC, si-EGR1-1#, or si-EGR1-2# (n=3). Data are shown as means ± SD; One-way ANOVA; ** p < 0.01.

**Table S1: siRNA target sequences**

| siRNA | Target sequence (5' to 3') |
| --- | --- |
| Negative Control | UUCUCCGAACGUGUCACGU |
| RNF187-1# | CACUGACCGACUACAAGAA |
| RNF187-2# | GGUUCAGGUCACUGCUGCA |
| WDR77-1# | GCAUGGAACGGCAGUUGGA |
| WDR77-2# | CUUGCUGUGCUGGACCUCAA |
| EGR1-1# | GCGGCAGAAGGACAAGAAA |

**Table S2: Plasmid information**

| Name | Function |
| --- | --- |
| pcDNA3.1-NC | negative control; empty vector |
| pcDNA3.1-Flag-RNF187 | plasmid expressing Flag-labeled RNF187 |
| pcDNA3.1-Flag-RNF187-MUT | plasmid expressing Flag-labeled RRNF187, the E3 ubiquitin ligase region of RNF187 is mutated |
| pcDNA3.1-Myc-WDR77-WT | plasmid expressing Myc-labeled WDR77 |
| pcDNA3.1-Myc-WDR77-K118R | plasmid expressing Myc-labeled WDR77, amino acid at position 118 mutated from lysine (K) to arginine (R) |
| pcDNA3.1-Myc- WDR77-K121R | plasmid expressing Myc-labeled WDR77, amino acid at position121 mutated from lysine (K) to arginine (R) |
| pcDNA3.1-Myc- WDR77-K179R | plasmid expressing Myc-labeled WDR77, amino acid at position 179 mutated from lysine (K) to arginine (R) |
| pcDNA3.1-Myc- WDR77-K201R | plasmid expressing Myc-labeled WDR77, amino acid at position 201 mutated from lysine (K) to arginine (R) |
| pRK5-HA-Ub | plasmid expressing HA-labeled wildtype Ub |
| pRK5-HA-Ub-K6 | Other lysine mutated to arginine, only expressing lysine-6 containing HA-labeled mutant Ub plasmid |
| pRK5-HA-Ub-K11 | Other lysine mutated to arginine, only expressing lysine-11 containing HA-labeled mutant Ub plasmid |
| pRK5-HA-Ub-K27 | Other lysine mutated to arginine, only expressing lysine-27 containing HA-labeled mutant Ub plasmid |
| pRK5-HA-Ub-K29 | Other lysine mutated to arginine, only expressing lysine-29 containing HA-labeled mutant Ub plasmid |
| pRK5-HA-Ub-K33 | Other lysine mutated to arginine, only expressing lysine-33 containing HA-labeled mutant Ub plasmid |
| pRK5-HA-Ub-K48 | Other lysine mutated to arginine, only expressing lysine-48 containing HA-labeled mutant Ub plasmid |
| pRK5-HA-Ub-K63 | Other lysine mutated to arginine, only expressing lysine-63 containing HA-labeled mutant Ub plasmid |

**Table S3: The primer sequences used in this study (5' to 3')**

| 18sRNA (gene expression) | Forward | AAACGGCTACCACATCCAAG |
| --- | --- | --- |
|  | Reverse | CCTCCAATGGATCCTCGTTA |
| RNF187 (gene expression) | Forward | GCTCTGTGAAGGCTGGAACT |
|  | Reverse | AGCGACTTGGATAAGGCTGG |
| WDR77 (gene expression) | Forward | GTCACTGGGCTGGTGTTCTC |
|  | Reverse | GGGACCAAGTCGCATCTCTC |
| EGR1 (gene expression) | Forward | CCCCGACTACCTGTTTCCAC |
|  | Reverse | TGGGTTTGATGAGCTGGGAC |
| GAPDH (ChIP-qPCR) | Forward | CACTCCTCCACCTTTGACGC |
|  | Reverse | CGCCAGACCCTGCACTTTTTA |
| EGR1 (ChIP-qPCR) | Forward | GCCGGTCCTGCCATATTAGG |
|  | Reverse | CCCGGATCCGCCTCTATTTG |

**Table S4: The antibodies information**

| Antigen | Species | Source | Catalog# | Dilution |
| --- | --- | --- | --- | --- |
| RNF187 | Novus Biologicals | Rabbit | NBP2-83456 | 1:1000 |
| WDR77 | Proteintech | Rabbit | 10115-1-AP | 1:750 |
| H4R3me2s | Proteintech | Rabbit | 61188 | 1:1000 |
| Flag-tag | SIGMA | Mouse | F1804 | 1:1000 |
| Myc-tag | Cell Signaling Technology | Mouse | 9402S | 1:1000 |
| HA-tag | Santa Cruz | Mouse | SC-7392 | 1:1000 |
| Ubiquitin | Santa Cruz | Mouse | Sc-8017 | 1:1000 |
| Ubiquitin-K48 | Abcam | Rabbit | ab140601 | 1:1000 |
| Tubulin | Proteintech | Mouse | 11224-1-AP | 1:3000 |
